# Watermelon Rind Dietary Fibers as Natural Source to Enhance Texture of Wheat Bread

**DOI:** 10.3390/foods13182909

**Published:** 2024-09-13

**Authors:** Molka Ben Romdhane, Amir Bouallegue, Mohammed Bourhia, Ali Bougatef, Ahmad Mohammad Salamatullah, Semia Ellouz-Chaabouni, Anissa Haddar

**Affiliations:** 1 Laboratory of Plants Improvement and Valorization of Agri-Resources, National School of Engineering of Sfax, University of Sfax, Sfax 3038, Tunisia; molkabenromdhane@yahoo.fr (M.B.R.); semia.chaabouniellouz@enis.tn (S.E.-C.); anissa.haddar@isbs.usf.tn (A.H.); 2 Laboratory of Food Oral Processing, School of Food Science and Biotechnology, Zhejiang Gongshang University, Hangzhou, 310018, China; amir.bouallegue@enis.tn (A.B.); 3 Department of Chemistry and Biochemistry, Faculty of Medicine and Pharmacy, Ibn Zohr University, Laayoune 70000, Morocco; bourhiamohammed@gmail.com; 4 High Institute of Biotechnology, University of Sfax, Sfax 3038, Tunisia; 5 Department of Food Science & Nutrition, College of Food and Agricultural Sciences, King Saud University, P.O. Box 2460, Riyadh 11451, Saudi Arabia; asalamh@ksu.edu.sa

**Keywords:** watermelon rinds, water-soluble polysaccharides, hemicellulose, bread formulation, alveograph analysis, bread quality

## Abstract

The objective of this study was to explore how watermelon rinds (WMRs) and their derivatives, specifically water-soluble polysaccharides (WMRPs) and hemicellulose (WMRH), as sources of dietary fiber, could enhance the quality of wheat bread. The extraction process yielded 34.4% for WMRP and 8.22% for WMRH. WMR, WMRP, and WMRH exhibited promising functional characteristics and were incorporated separately into wheat flour with low bread-making quality (FLBM) at varying proportions (0.5%, 1%, and 1.5% (*w*/*w*)). The volume, texture, and crust and crumb color underwent evaluation and were compared to the control. The findings indicated that incorporating WMR notably enhanced the alveograph profile of the dough, demonstrating a more effective impact than the addition of WMRP and WMRH. Adding WMR, WMRP, and WMRH at a 1% concentration to low-quality wheat flour for bread making increased the deformation work values by 16%, 15%, and 13%, respectively, and raised the P/L ratios by 42%, 36%, and 38%, respectively. Additionally, the assessment of the bread highlighted a substantial enhancement in both volume and texture profile when WMR was added, in contrast to the control bread (made with FLBM). These findings underscore that incorporating 1% WMR into FLBM was the most effective means of improving bread quality based on the results of this study.

## 1. Introduction

The preparation and consumption of fruits and vegetables generate substantial volumes of solid waste from agricultural sources. These waste materials carry the risk of environmental pollution, leading to the depletion of valuable biomass and nutrients. Indeed, agricultural and industrial residues serve as appealing reservoirs of polyphenols, natural antioxidants, and dietary fibers [1,2]. Given the significance of these residues as important sources of functional ingredients, their valorization emerges as a promising field to explore.

Watermelon is one of the most widely consumed fruits globally, with a total yield of approximately 117 million tons [3]. However, 30–50% of its weight is often discarded as watermelon rind (WR) [4]. Watermelon grown in the warm parts of the world is usually not suitable for processing and is mostly used for direct consumption [5]. During hot and dry summers, this fruit is very attractive to people on account of its cool, refreshing, and pleasing taste and attractive red color. Around half of a watermelon is typically edible, while the other half comprises over 35% rind and 15% peel is usually discarded [6]. But the watermelon rinds have an interesting nutritional value and can be exploited in different domains. In fact, the research by Al-Sayed and Ahmed [7] and Pires et al. [8] highlights the significance of watermelon by-products as crucial reservoirs of protein, dietary fibers, and natural antioxidants. Watermelon (*Citrullus lanatus*) is a fruit that belongs to the Cucurbitaceae family [9,10]. Based on United States Department of Agriculture (USDA) data [11], every 100 g of watermelon consists of 92.0% water, 7.6% carbohydrates with 6.2% as sugars, 0.4% dietary fiber, 112 mg of potassium, 10 mg of magnesium, 8.1 mg of vitamin C, 4.5 mg of lycopene, and 0.3 mg of β-carotene (a vitamin A precursor) and supplies 30 kilocalories. Recently, Cristo et al. [12] reported that watermelon is an excellent source of vitamin C and vitamin A and contains a significant amount of vitamin B6, vitamin B1, and minerals, like iron, potassium, magnesium, and phosphorus. Additionally, watermelon serves as a significant source of natural antioxidants, like carotenoids, phenolic compounds, and essential amino acids, such as citrulline and arginine [13,14]. Citrulline, initially discovered in watermelon juice, possesses promising antioxidant properties and plays a role in vasodilation [15]. Rimando and Perkins-Veazie [14] have noted that watermelon rinds boast substantial citrulline content, suggesting the potential for the creation of a valuable product out of agricultural waste. Additionally, citrulline’s conversion into arginine, a vital amino acid crucial for heart health, the circulatory system, and immune function, led the researchers to speculate that watermelon rinds could potentially relax blood vessels, contributing to the prevention of diseases, like cancer and cardiovascular disorders.

The industrial processing of watermelon rinds offers a promising opportunity to convert this agricultural waste into valuable products. This approach not only helps reduce waste but also meets the increasing demand for sustainable and eco-friendly solutions. To scale this up, optimizing the processes and managing the supply chain are essential to ensure a steady supply of raw materials and cost-effective production. Financial success depends on a thorough cost analysis, a market demand assessment, and an understanding of the return on investment. Profitability can be improved by diversifying revenue streams, achieving economies of scale, and aligning with market trends in the food, pharmaceutical, and sustainable sectors.

Nowadays, the demand for wheat-based products with functional ingredients added (higher amounts of dietary fibers and antioxidants) is growing rapidly in accordance with an increasing number of consumers who prefer to eat healthier foods in their daily diet in order to prevent diseases [16]. This motivates the food industry and researchers to focus on optimizing bread-making technology, aiming to enhance the quality, taste, and availability of food products like bread [17]. Bread, the most widely consumed bakery product globally, is crafted from wheat flour, a significant source of carbohydrates, but it is deficient in several other essential components, like minerals and dietary fibers [18]. So, in order to ensure adequate dietary fiber intake in our daily diets, it is crucial to develop innovative enriched wheat-based products, like dietary fiber and mineral-fortified bread, to meet these nutritional requirements. To augment the fiber levels in bread, various raw materials, like date seeds [19], watermelon rind [6], carob seeds [20], and almond gum [21], have been incorporated. Among these, watermelon rind powder emerges as a promising fiber source, particularly for bread and other food applications. Thus, the objective of this study was to explore the impact of utilizing watermelon rinds (WMRs), water-soluble polysaccharides (WMRPs), and hemicellulose (WMRH) extracted from WMRs on bread formulation. The properties of wheat flour dough’s alveograph were investigated. Additionally, research was conducted on how WMR and its derivatives affected the volume, color, and textural characteristics of the designed bread when used as bread additives.

## 2. Materials and Methods

### 2.1. Wheat Flour and Watermelon Rinds

The commercially acquired flour of low bread-making quality (FLBM) contained 10.75% moisture, 0.36% ash, and 8.4% protein content. Meanwhile, the flour of high bread-making quality (FHBM) served as the reference, with 13.19% moisture, 0.33% ash, and 8.73% protein content. The flours of low and high bread-making quality were provided by a local Tunisian company that produces durum wheat semolina and soft wheat flour, and the characterization of these flours was released by this company.

The fruits utilized in this study were sourced from the local market in Sfax, Tunisia. The discarded material employed was derived from watermelon (*Citrullus lanatus*) rinds, referred to as WMRs. The watermelon rind was separated from the washed fresh watermelon fruits and cut into small pieces with a knife. After weighing the watermelon rind, the pieces were dehydrated at 50 °C until thoroughly dried. The dried rind was then ground to a particle size between 500 and 800 µm and stored for further use.

### 2.2. Chemical Characterization of Watermelon Rinds

Dry matter was assessed according to Cunni’s method [22]. The total nitrogen content was measured using Kjeldahl’s method [23], and protein content was derived using the standard factor of 6.25. Fat and total carbohydrate were determined as per the methods specified by the French Association for Standardization [24] and Dubois et al. [25], respectively.

The ash content was determined by combusting the samples in a muffle furnace at 550 °C for 4 h. The mineral composition was then assessed using the atomic absorption spectrophotometer ZEEnit 700 (Analytik Jena, Saint-Aubin, France).

The dietary fiber content was determined using the enzymatic–gravimetric method [26]. Additionally, the insoluble lignin content in the watermelon rinds powder was determined by subjecting it to acid hydrolysis, followed by filtration, drying, and weighing, following the Technical Association of Pulp and Paper Industry (TAPPI) T222 om-11 standard method [27].

The holocellulose content (cellulose + hemicellulose) was assessed using the sodium chlorite method, following the TAPPI T257 om-09 standard [28]. The cellulose content was subsequently determined by subjecting the extracted holocellulose to hydrolysis with a sodium hydroxide solution, as per the TAPPI T257 om-09 standard. To calculate the hemicellulose content, the amount of cellulose was subtracted from the holocellulose content.

### 2.3. Extraction of Soluble Polysaccharides and Hemicellulose from Watermelon Rinds

**Figure 1:**
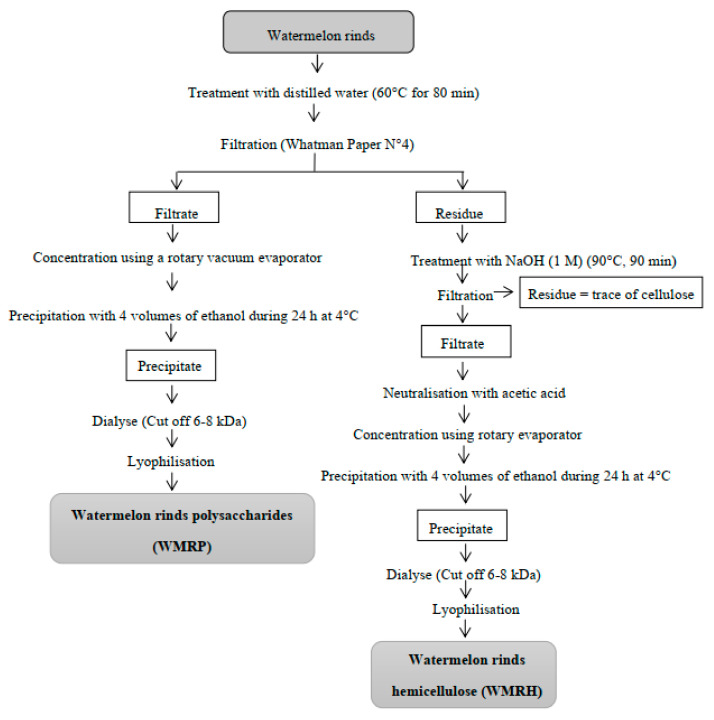
Extraction steps of the water-soluble polysaccharides (WMRPs) and hemicellulose (WMRH) from the watermelon rinds.

The powdered watermelon rinds were fractionated into water-soluble polysaccharides (WMRPs) and hemicellulose (WMRH) according to Yao et al. [29] and Peng et al. [30], respectively, with slight modifications (Figure 1). The watermelon rind powder was diluted 20 times in distilled water and then subjected to incubation in a thermostatic water-bath at 60 °C for 80 min. The resulting aqueous extract was filtered through Whatman no. 4 filter paper, resulting in two fractions: the filtrate and the residue. These fractions were employed in preparing the WMRP and WMRH, respectively. The filtrate was concentrated initially using a rotary vacuum evaporator (Shanghai, China) at 50 °C. Subsequently, the WMRPs (water-soluble polysaccharides) were precipitated by adding 4 volumes of ethanol (95%) to the concentrated filtrate, which was then incubated at 4 °C for 24 h. The resulting precipitate was collected through centrifugation (3000× *g*, 15 min), dissolved in deionized water, and subjected to a 3-day dialysis process (cut-off = 6–8 kDa). Finally, the obtained WMRP was freeze-dried into a powdered form. The yield of the recovered WMRP (%, *w*/*w*) was calculated using Equation (1).

(1) $$\mathrm{WMRP}\;\mathrm{recovery}\;\mathrm{yield}\;\left(\%\right)=\left(\frac{\mathrm{Lyophilised}\;\mathrm{WMRP}\;\mathrm{weight}\;\left(g\right)}{\mathrm{Watermelon}\;\mathrm{rinds}\;\mathrm{pouder}\;\left(g\right)}\right)\times 100$$

The residue was used to prepare the WMRH. To achieve this, it underwent incubation in a thermostatic water bath at 90 °C for 90 min in the presence of 500 mL of NaOH solution (1 M). Afterwards, the obtained extract was filtered through Whatman n° 4 filter paper. Upon neutralization, the recovered supernatant was concentrated using a rotary vacuum evaporator at 50 °C. The WMRH was subsequently precipitated by adding 4 volumes of ethanol (95%) and left to incubate at 4 °C for 24 h. The resulting precipitate was retrieved through centrifugation (3000× *g*, 15 min) and then dissolved in deionized water. Following a 3-day dialysis step (cut off = 6–8 kDa), WMRH powder was obtained through lyophilization. The recovery yield of the WMRH (%, *w*/*w*) was calculated using Equation (2).

(2) $$\mathrm{WMRH}\;\mathrm{recovery}\;\mathrm{yield}\;\left(\%\right)=\left(\frac{\mathrm{Lyophilised}\;\mathrm{WMRH}\;\mathrm{weight}\;\left(g\right)}{\mathrm{Watermelon}\;\mathrm{rinds}\;\mathrm{pouder}\;\left(g\right)}\right)\times 100$$

### 2.4. Physicochemical Analyses of WMR Powder and WMR Fractions

#### 2.4.1. FT-IR Spectroscopic Analysis

The FT-IR spectrum of the WMR powder, WMRP, and WMRH was obtained utilizing a Fourier transform infrared spectrophotometer (Perkin Elmer Spectrum BX FT-IR, Shelton, CT, USA). The sample was ground and mixed with spectroscopic-grade potassium bromide (KBr) powder and subsequently pressed into 1 mm disks. The results were recorded within the wavenumber range of 4000–400 cm^−1^ [31].

#### 2.4.2. Scanning Electron Microscope

The WMR powder, WMRP, and WMRH were gold-coated using a sputter coater (JFC-1100, JEOL, Tokyo, Japan) and subsequently examined by an SEM system (JSM-5400, JEOL, Tokyo, Japan) under high vacuum conditions.

#### 2.4.3. Color Measurement

The color attributes of the WMR powder, WMRP, and WMRH were determined by assessing the CIE (International Commission on Illumination) Lab coordinates (L*, a*, b*) using a Mini Scan XETM spectrophotocolorimeter manufactured by Hunter Lab Inc., Reston, VA, USA. In this system, L* represents lightness, ranging from black (0) to white (100); a* spans from green (−) to red (+); and b* extends from blue (−) to yellow (+). The findings were presented as the mean of five measurements obtained from various points on the samples, all taken at room temperature. The measurements were conducted using a standard white plate (with L* = 93.68, a* = 0.69, and b* = 0.88) as the reference.

#### 2.4.4. Water Activity

The water activity (aw) was determined at 25 °C using a Novasina aw Sprint TH-500 Apparatus (Pfaffikon, Zurich, Switzerland), which featured automatic calibration for measurements across 0.04 to 0.98 aw, with up to 8 calibration points.

### 2.5. Functional Proprieties

#### 2.5.1. Water-Holding Capacity

The water-holding capacity (WHC) was determined following a partially modified procedure based on that of Lin et al. [32]. Firstly, 0.5 g of WMR or its fraction WMRH was placed into a pre-weighed centrifuge tube. Subsequently, 50 mL of distilled water was added to the tube and left at room temperature for 60 min. The solutions were intermittently mixed for 5 s every 15 min for a duration of 1 h. Afterwards, the solutions underwent centrifugation at 15,025× *g* for 20 min using a refrigerated centrifuge (Hettich Zentrifugen, ROTINA 380R, Schwerte, Germany). Following centrifugation, the upper phase was removed, and the centrifuge tube was tilted to a 45° angle, allowing drainage for 30 min on a filter paper. The water-holding capacity was calculated as the grams of retained water per gram of sample on a dry basis, employing Equation (3).

(3) $$\mathrm{WHC}\;\left(g\;\mathrm{water}/\mathrm{dry}\;\mathrm{sample}\right)=\left(\frac{\mathrm{Weight}\;\mathrm{of}\;\mathrm{the}\;\mathrm{tube}\;\mathrm{content}\;\mathrm{after}\;\mathrm{draining}\;\left(g\right)-\mathrm{weight}\;\mathrm{of}\;\mathrm{dried}\;\mathrm{sample}\;\left(g\right)}{\mathrm{Weight}\;\mathrm{of}\;\mathrm{dried}\;\mathrm{sample}\;\left(g\right)}\right)$$

#### 2.5.2. Fat-Binding Capacity

The fat-binding capacity (FBC) was assessed following Lin et al.’s method [32] with slight modifications. Each watermelon rind sample (0.5 g of WMR and WMRH) was combined with 10 mL of soybean oil and left at room temperature for 60 min (agitated briefly for 5 s every 15 min). Subsequently, the mixture was centrifuged at 5869 g for 20 min, and the supernatant was discarded. The centrifuge tube was then tipped at a 45° angle and left to drain on a filter paper for 30 min. The FBC was calculated using Equation (4).

(4) $$\mathrm{WHC}\;\left(g\;\mathrm{oil}/\;\mathrm{dry}\;\mathrm{sample}\right)=\left(\frac{\mathrm{Weight}\;\mathrm{of}\;\mathrm{the}\;\mathrm{tube}\;\mathrm{content}\;\mathrm{after}\;\mathrm{draining}\;\left(g\right)-\mathrm{weight}\;\mathrm{of}\;\mathrm{dried}\;\mathrm{sample}\;\left(g\right)}{\mathrm{Weight}\;\mathrm{of}\;\mathrm{dried}\;\mathrm{sample}\;\left(g\right)}\right)$$

#### 2.5.3. Foaming Proprieties

The foaming capacity (FC) and foam stability (FS) of the WMR and WMRH were assessed following Shahidi et al.’s described method [33]. Various concentrations of WMR, and WMRH (1%, 2%, 4%, and 5% (*w*/*v*)) were prepared in 20 mL of water. Homogenization for 2 min at room temperature (20 °C) was conducted using a Moulinex R62 homogenizer (Organotechnie, Courneuve, France) to incorporate air. The whipped samples were promptly transferred into a graduated cylinder, and the total volume was instantly measured after whipping. Foam expansion was calculated as the percentage of volume increase after homogenization at 0 min and was determined using the following equation (Equation (5)):

(5) $$\mathrm{FC}\;\left(\%\right)=\left(\frac{V_{T}-{\;V}_{0}}{V_{0}}\right)\times 100$$

Foam stability was measured as the volume of foam remaining after 10 min, 20 min, 30 min, and 60 min.

$$\mathrm{FS}\;\left(\%\right)=\left(\frac{V_{t}-{\;V}_{0}}{V_{0}}\right)\times 100$$

where V_T_ is the total volume after whipping (mL); V_0_ is the volume before whipping and Vt is the total volume after leaving at room temperature (10–60 min). All the determinations are the means of at least three measurements.

#### 2.5.4. Emulsifying Activity and Emulsion Stability

The emulsifying properties of the samples were evaluated using a partially modified turbidimetric method [34]. Fifty milliliters of each suspension at different concentrations (1, 2, 3, 4, and 5%; *w*/*v*) was mixed with 10 mL of soybean oil and homogenized for 3 min at 20 °C. Samples of the emulsion (50 µL) were immediately extracted from the bottom of the container and, after 10 min, diluted with 5 mL of 0.1% sodium dodecyl sulfate (SDS) solution. Subsequently, the absorbance of the diluted emulsions was measured at 500 nm using a spectrophotometer (UV mini 188 1240, UV/VIS spectrophotometer, SHIMDZU, Beijing, China). The emulsifying capacity was established based on the absorbance measured right after emulsion formation. Subsequently, the emulsion activity index (EAI) was determined using Equation (6):

(6) $$EAI\;\left(m^{2}/g\right)=\frac{2\times 2.303\times A_{0}\;}{\varphi \times sample\;weight\;\left(g\right)}$$

where A_0_ is the absorbance measured immediately at 500 nm, and φ presents the oil volumetric fraction (0.25). The emulsion stability (ES) was evaluated as (Equation (7)):

(7) $$ES\;\left(\%\right)=\frac{A_{10}}{A_{0}}\times 100$$

where A_0_ presents the initial absorbance, and A_10_ is the absorbance measured at 10 min after emulsion formation.

### 2.6. Dough Preparation and Characterization

The dough was created by blending these components: 300 g of wheat flour, 4.8 g of sucrose, 5.75 g of sodium chloride, 3 g of wet compressed yeast (*Saccharomyces cerevisiae*), 12 mL of soybean oil, and 195 mL of water. After kneading and fermentation, the dough was baked for 3 h in a bread maker (Home Carrefour HBM1228, Massy, France). The various ingredients were initially placed in the bread pan and kneaded for 14 min. The resulting dough underwent a 20 min fermentation before a second 20 min kneading. Subsequently, the dough rested undisturbed at room temperature for 30 min following kneading. Dough made with high-quality bread-making wheat flour (FHBM) was employed as the positive control, while dough made with low-quality bread-making wheat flour (FLBM) served as the negative control. The FLBM was supplemented with varying amounts (0.5%, 1%, and 1.5%) of WMR, WMRP, and WMRH to examine their influence on the textural and nutritional aspects of bread.

The alveograph properties of the dough samples were assessed using a Chopin alveograph (Châtillon, France). A computer program developed by R Design company (Pullman, Washington, DC, USA) automatically recorded the following parameters: maximum overpressure (P), representing resistance to extension; average abscissa (L) at bubble rupture, indicating dough extensibility; deformation energy (W), reflecting dough strength; and the P/L ratio (elastic resistance and extensibility balance of the flour dough), providing insight into the dough quality.

### 2.7. Bread Making and Characterization

#### 2.7.1. Bread Making

The bread baking process took place in a bread maker’s bread pan (Home Carrefour HBM1228) for 60 min. Following baking, the bread samples were allowed to cool for 30 min before undergoing further characterization.

#### 2.7.2. Bread Volume

The bread volume and weight were assessed following the method outlined by Yi et al. [35]. Once baked and cooled, each bread sample from the respective mixture was initially positioned within a 2 L beaker, the volume of which was known as VC. The container was then filled with rapeseed until the bread was displaced, allowing the volume of rapeseed, VR, to be measured. The discrepancy between the container volume and the rapeseed volume, VC–VR, determined the bread volume, VB. The bread weight, WB, was subsequently measured, and the bread’s specific volume (VS) was calculated using Equation (8):

(8) $$V_{S\;}\left({\mathrm{cm}}^{3}/g\right)=\frac{V_{B}}{W_{B}}$$

#### 2.7.3. Bread Textural Analysis

For every bread sample, the texture was assessed using the texture profile analysis (TPA) test. A texturometer (Lloyd Instruments Ltd., Bognor Regis, UK) with a 35 mm diameter cylindrical aluminum probe was used. The texture analyzer was connected to a computer, which used software from Texture Technologies Corp. (Scarsdale, NY, USA) to evaluate the data and adjust the parameters. After compressing the bread pieces to 40% of their initial height, measurements were taken on sections measuring 2 cm in width, 4 cm in length, and 5 cm in height. The settings used were 0.5 mm × s^−1^ speed and 5 s intervals between consecutive compressions. Slices of bread were positioned vertically beneath the probe. The fundamental characteristics of hardness, cohesiveness, and springiness, as well as the secondary mechanical characteristic of adhesion, were determined using the textural curves generated for each variety of bread [36]. All the texture assessments were conducted three times for each bread specimen. In addition to primary parameters and secondary mechanical traits, the bread texture analyses were conducted after the bread had cooled to room temperature (approximately 2 h post-baking) [37].

#### 2.7.4. Bread Color Evaluation

The crumb and crust colors of the bread were assessed using a tri-stimulus colorimeter (HunterLab Inc., Reston, VA, USA) employing the CIE Lab scale (L*, a*, and b*), following established procedures. The recorded bread color values were the mean of five measurements taken at room temperature from distinct locations within each sample.

### 2.8. Statistical Analysis

The experiments were conducted in triplicate, and the results were presented as mean values with their corresponding standard deviations. Statistical analysis was performed using Student’s *t*-test and Duncan’s multiple range test (SPSS software 17.0) to determine significant differences between groups. A significance level of *p* < 0.05 was considered to indicate statistical significance.

## 3. Results

### 3.1. Chemical Composition of Watermelon Rinds

Based on the phytochemical composition, watermelons contain detectable levels of carbohydrates, saponins, glycosides, steroids, alkaloids, polyphenols, flavonoids, and tannins, and also produce various by-products such as rinds [38]. The analysis of the raw material’s chemical composition (WMR), as revealed in Table 1, demonstrated that carbohydrates constituted the largest portion (66.00 ± 2.2%). Notably, the WMR displayed significant levels of protein (17.00 ± 0.13%) and minerals (2.00 ± 0.56%). Conversely, the WMR exhibited a comparatively low-fat content (2.00 ± 0.14%). These findings align with the earlier discoveries made by Al-Sayed and Ahmed [5], indicating that their watermelon rinds consisted of 10.61% moisture, 56.02% carbohydrates, 11.17% protein, 2.44% fat, and 13.09% ash. Similarly, a notable amount of dietary fiber, totaling 55%, comprising both soluble and insoluble fiber, was observed. The dietary fibers within the raw material (WMR) primarily consisted of water-insoluble polysaccharides, hemicellulose, and cellulose. The analysis revealed that the water-soluble polysaccharide content was 9.00 ± 0.72%. Higher quantities of hemicellulose and cellulose were recorded at 25.00 ± 2.3% and 21.00 ± 0.30%, respectively. The mineral composition analysis revealed that the watermelon rinds were notably abundant in potassium, calcium, sodium, and magnesium, with respective contents of 601 mg/100 g ± 17, 346 mg/100 g ± 12, 287 mg/100 g ± 10, and 124 mg/100 g ± 6.

### 3.2. Extraction Yields of WMRP and WMRH

In an earlier investigation, Ben Romdhane et al. [39] recorded a notable extraction yield of polysaccharides (WMRPs) at 34.4% ± 1.72 from WMR. This was attained using optimized conditions: maintaining a solvent to raw material ratio of 10 mL/g, an extraction duration of 80 min, and a temperature of 60 °C. As depicted in the extraction diagram delineating WMRP and WMRH (Figure 1), the residual solid subsequent to extracting the water-soluble polysaccharides underwent an alkaline extraction process to procure the insoluble polysaccharides or hemicelluloses. The extraction yield for the hemicelluloses was approximately 8.00% ± 0.41. Hemicelluloses represent a category of plant cell wall polysaccharides interconnected with other cell wall constituents, like cellulose, proteins, lignin, and phenolic compounds, through various bonds, including covalent, hydrogen, ionic, and hydrophobic interactions. Hemicelluloses are recognized as heterogeneous polysaccharides, diverging in biosynthetic pathways from cellulose [40].

### 3.3. FT-IR Spectroscopy Analysis of WMR, WMRP, and WMRH

**Figure 2:**
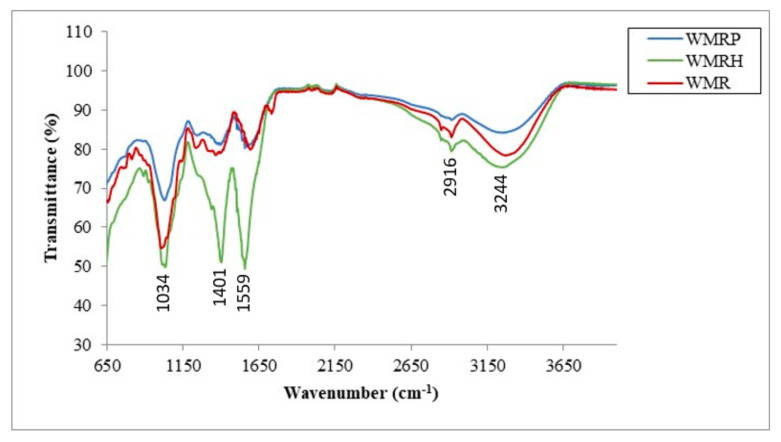
FT-IR spectra of watermelon rinds, water-soluble polysaccharides (WMRPs) and hemicellulose (WMRH).

Infrared spectroscopy analysis (Figure 2) was employed to examine the functional groups present in WMR, WMRP, and WMRH. The findings revealed characteristic transitions in the ranges of 3000–3700, 1500–1770, and 950–1200 cm^−1^ for WMR and its derivatives. These transitions are indicative of the presence of polysaccharide structures [29,41]. The wide band spanning from 3000 cm^−1^ to 3700 cm^−1^ indicated the characteristic peak associated with the stretching vibration of hydrogen-bonded O-H groups. The minor band observed at 2916 cm^−1^ was linked to C-H absorption, encompassing the stretching and bending vibrations of CH, CH2, and CH3 groups. These spectral features align with the characteristic absorption patterns of polysaccharides, as previously noted [41]. Upon further examination of the FT-IR spectrum, it was observed that the signals at 1735 cm^−1^ indicated the presence of the carbonyl group, while the ranges of 1011–1034 cm^−1^ and 1235 cm^−1^ were associated with the C-O and C-O-C groups, confirming the existence of pyranose rings [42,43,44]. Additionally, the band observed between 1559 and 1595 cm^−1^ was attributed to the vibration related to N-H deformation.

Due to the structural similarities between cellulose, hemicelluloses, and soluble polysaccharides, some of the FT-IR spectral bands may overlap or appear to be common across different fractions. Despite this overlap, it is still possible to assign distinct spectra to specific fiber fractions within the samples. The FT-IR spectra for the soluble (WMRP), insoluble (WMRH), and watermelon rind powder (WMR) fractions were analyzed and reported. The intensity of the main bands can be correlated with the amount of insoluble fiber present in each sample, with the order of WMRH > WMR > WMRP, which aligns with the results obtained from chemical analysis. Although all the samples exhibited similar overall spectral profiles, the main bands occurred at the same wavelengths but with varying intensities. Notably, the bands at 1034 cm^−1^, 1401 cm^−1^, 1559 cm^−1^, 2916 cm^−1^, and 3244 cm^−1^ were present across all the samples, highlighting their consistent spectral features despite differences in insoluble fiber content.

### 3.4. Scanning Electron Microscope

**Figure 3:**
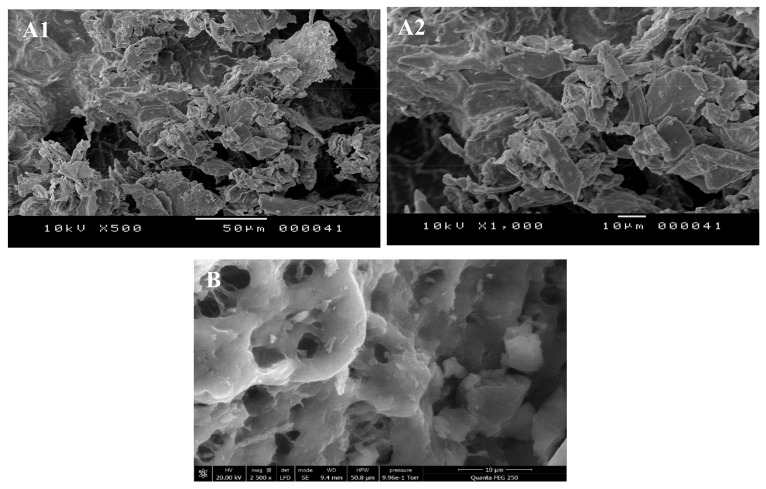
SEM micrographs of WMR and WMRH with different magnifications: WMR (50, 10 μm, ×500 (**A1**), ×1000 (**A2**), 10 kV) and (**B**) WMRH (10 μm, ×2500, 10 kV).

SEM analysis was employed to explore the microstructure of the WMR and WMRH, and the corresponding images are displayed in Figure 3. The findings indicated that the raw material WMR exhibited a rough surface (Figure 3(A1,A2)). Similarly, the water-soluble polysaccharides (WMRPs) showcased a rough surface with numerous cavities [39], while the microstructure of the alkaline-soluble fraction (WMRH) appeared more delicate, displaying a smoother surface (Figure 3B).

The variation in structure can be attributed to the extraction conditions applied to each saccharide fraction. The conditions for the WMRH involved more aggressive parameters (alkaline conditions and relatively high temperature), potentially leading to partial degradation of this fraction. Both saccharide fractions exhibited a porous microstructure in terms of porosity. In contrast to Cheng et al.’s findings [45], where *Epimedium acunatum* polysaccharides extracted via hot water displayed a flat and smooth surface, these results indicated diverse morphologies among the materials. These differences could potentially influence distinct physical characteristics, like solubility, water/oil-holding capacities, and emulsion properties [46].

### 3.5. Color Measurement and Water Activity

Table 2 displays the color parameters and water activity of the WMR, WMRP, and WMRH. Water activity signifies the quantity of unbound water present in materials, which potentially enables biological or chemical spoilage. Its significance extends across diverse industries, such as food, pharmaceuticals, cosmetics, seed storage, etc. Maintaining the product’s water activity below a specific threshold is crucial to ensure microbiological and physicochemical stability [47]. The water activity (aw) values measured were 0.30 ± 0.02 for WMR, 0.40 ± 0.02 for WMRP, and 0.30 ± 0.06 for WMRH. These values, as indicated by the water activity stability map, align with low lipid oxidation, slow non-enzymatic browning (Maillard reactions), and a reduced risk of microbial growth. Color holds significant importance as a quality parameter for dried powders, influencing the suitability of an additive for its intended application. In food processing involving polysaccharides, it is essential to steer clear of unusual colors and off-flavors. Table 2 showcases the CieLab coordinates (L*, a*, and b*) for the raw materials WMR, WMRP, and WMRH. The findings revealed relatively low L* values, spanning from 40 to 45 for WMRH and WMRP. The watermelon rinds displayed the highest luminosity with a value of L* = 80.00 ± 0.13. These observations diverged from those of Ben Jeddou et al. [48], where polysaccharides extracted from potato peels exhibited a lighter shade (L* = 82.00 ± 0.02) compared to the WMR fractions. Notably, the soluble polysaccharides (WMRPs) demonstrated notably higher red (a*) and yellow (b*) colors (a* = 7.50 ± 0.12; b* = 15.50 ± 0.55) compared to the insoluble fraction (WMRH) (a* = 4.00 ± 0.2; b* = 7.00 ± 0.06); this was a difference that reached statistical significance (*p* < 0.05). Ben Jeddou and colleagues [47] indicated that the red and yellow color values of the polysaccharides extracted from potato peels were recorded as a* = 3.35 ± 0.01 and b* = 13.60 ± 0.08, respectively. The differences in color are indicative of the distinct starting raw materials and merit attention in their respective applications [49]. El Ksibi et al. [50] highlighted that a majority of solid organic agricultural waste materials derive their color from compounds like antioxidants, antimicrobials, vitamins, or pigments, all carrying potential nutritional or functional properties.

The color differences between watermelon polysaccharides and fibers can be attributed to their distinct molecular compositions and the effects of the extraction and processing methods. Polysaccharides tend to be lighter with a more neutral hue, while fibers, especially after processing, exhibit a darker and redder color. As bread production progresses, particularly through steps like mixing and heating, these color changes become more pronounced, leading to a darker, more red-hued product. In contrast, wheat flour remains the lightest and least red, highlighting the significant impact of these processes on the final bread color.

### 3.6. Functional Proprieties of WMR, WMRP, and WMRH

#### 3.6.1. Water-Holding and Fat-Binding Capacities

Water-holding and fat-binding abilities represent functional properties linked directly to texture through interactions among components, like water and oil. The water-holding capacity (WHC) and fat-binding capacity (FBC) of WMR and WMRH were examined. As indicated in Table 3, the WMR exhibited a notable water-holding capacity (12.00 ± 0.3 g H_2_O/g sample), which was significantly (*p* < 0.05) higher compared to the same values of the WMRP and WMRH (2.00 ± 0.2 g H_2_O/g sample).

The watermelon rinds (WMRs) exhibited a higher WHC value compared to that recorded in both pea flour (3.69 g/g) and broad bean flours (4.46 g/g) [51]. However, this finding aligns with the values reported by Grigelmo-Miguel et al. [52], who analyzed the WHC of orange and peach fibers, noting respective values of 12.4 g/g and 12.6 g/g. Additionally, the WHC value recorded for the WMR exceeded those documented for grapefruit, lemon, orange, and apple by-products (1.6–2.3 g/g) [53], as well as the recently reported WHC values for diverse by-products from different botanical origins by Masli et al. [54], ranging from 1.3 to 3.9 g/g. Notably, it even surpassed the WHC values observed for wheat bran (2.7–3.6 g/g) [55]. The high value of WHC indicates the potential utilization of WMR in products needing hydration for viscosity or texture enhancement [56]. Additionally, it could be beneficial in physical stress production processes, like kneading, extrusion, and homogenization. Furthermore, it aids in preventing syneresis phenomena during storage [54]. The water-holding capacities of the WMRP (2.00 ± 0.1 g/g) and WMRH (2.00 ± 0.1 g/g) samples were lower than those found for galactomannans (15.20 g/g) [57], but they closely resembled the values reported by Sila et al. [58] for the soluble polysaccharides extracted from the by-products of pistachio juice production (1.46 g/g) and almond barley production (1.95 g/g). In terms of OHC, the measured values for WMR (2.00 ± 0.1 g oil/g sample), WMRP (4.00 ± 0.1 g oil/g sample), and WMRH (4.00 ± 0.1 g oil/g sample) exceeded those reported by Belghith-Fendri et al. [51] for pea and bean pod flours, which stood at values of 1.14 g/g and 1.42 g/g, respectively. Moreover, they surpassed the values found for the polysaccharides of pea (0.28 g/g) and broad bean (0.13 g/g) [59]. They also exceeded the levels of cauliflower fiber (0.50 g/g) [60] and wheat bran (1.50 g/g) [61]. The hydrophobic nature of the fiber particles, their capacity to adsorb organic compounds onto their surface—which is correlated with their chemical composition—and the porosity and affinity of the fiber molecules’ structure for oil are all factors that contribute to the significance of the OHC feature in polysaccharides [56,62]. The discovered values indicate the potential use of WMR, WMRP, and WMRH in formulating emulsions and high-fat food products, enhancing their physicochemical stability and aroma retention.

#### 3.6.2. Foaming Properties

**Figure 4:**
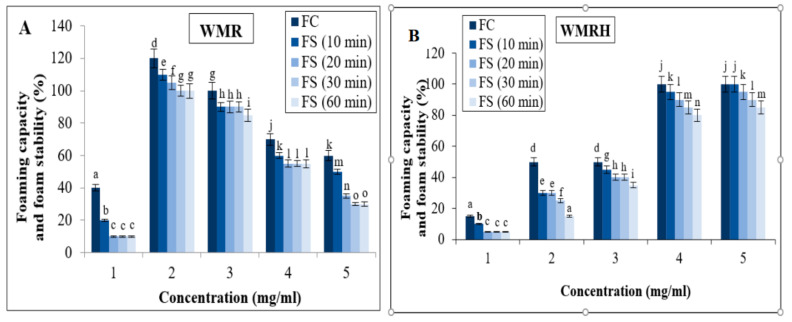
Foaming capacity and stability of the WMR (**A**) and WMRH (**B**) at different concentrations. Each value represents the average of three biological replicates ± SD. Same letter above the bars shows the statistical differences between the tested samples and the standard.

The ability to foam relies on surface characteristics, determined by both size and stability. Proteins and polysaccharides among various food macromolecules play an important role in stabilizing foam [63]. The formation and stability of foam are greatly influenced by the interfacial characteristics of the surface-active components employed in the formulation [64]. The foaming characteristics (foaming capacity (FC) and foam stability (FS)) of the WMR (Figure 4A) and WMRH (Figure 4B) are assessed across various concentrations (1%, 2%, 3%, 4%, and 5% (*w*/*v*)). The results show that as the sample concentration increased, the FC also increased proportionally, reaching peak values at a 2% concentration for the WMR (120 ± 1.75%) and 4% for the WMRH (100 ± 2.87%) and WMRP (150 ± 3.08%) [39]. This pattern indicates the potential of these polysaccharides to enhance foam production, leading to smaller, denser bubbles and increased gas retention within the foam. Regarding the stability of the created foam, there is a slight decrease in foam volume within the concentration range of (2–5%) after 10 min for the various samples. Beyond this time, the foam produced shows almost no change across all the concentrations tested. The interesting FC and FS values observed in WMR, WMRP [39], and WMRH indicate their capability to augment solution viscosity and create a system that stabilizes the gas–liquid interfacial film. Therefore, WMR, WMRP, and WMRH might hold potential for the enhancement of functional properties in diverse food formulations, such as ice cream, milkshakes, and marshmallows [65].

#### 3.6.3. Emulsifying Properties

**Figure 5:**
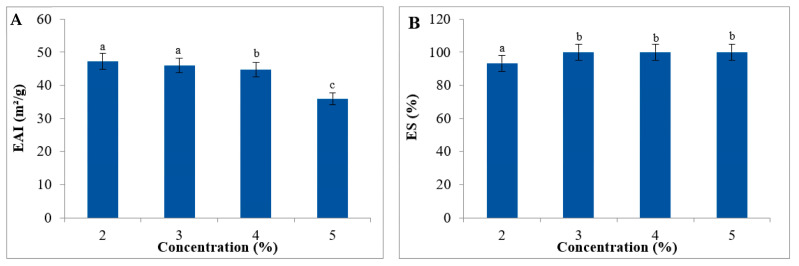
Emulsion capacity (**A**) and stability (**B**) of the WMRH at different concentrations. Each value represents the average of three biological replicates ± SD. Same letter above the bars shows the statistical differences between the tested samples and the standard.

An emulsion is characterized as a blend of two or more typically immiscible liquids. Figure 5 illustrates the emulsifying capacity (EC) and emulsion stability (ES) of the WMRH across different concentrations (1%, 2%, 3%, 4%, and 5%; *w*/*v*). At a 4% concentration, the WMRH exhibits its peak EC value (59.00 ± 0.97 m^2^/g) (Figure 5A). The emulsifying capacity (EC) and emulsion stability (ES) values of the WMRH were lower than those of the WMRP across all the concentrations [39]. The results acquired illustrate the remarkable emulsifying capacity of derivatives from watermelon rinds. In terms of stability, the emulsions formulated with the WMRH displayed consistent stability, progressively increasing with higher sample concentrations (Figure 5B). This finding indicates that the effectiveness of polysaccharides relies on their concentration within the aqueous phase of the emulsion [66]. Certainly, the acquired results highlight the intriguing emulsifying characteristics of WMRP [39] and WMRH, suggesting their promising potential as additives in the food industry. The formation of emulsions might be linked to the favorable surface properties exhibited by these two fractions. Studies have shown that macromolecular emulsifiers undergo conformational changes, potentially increasing the number of interfacial contacts per molecule. This process elevates the effective concentration of the adsorbed segments and consequently reduces interfacial tension [67]. Hence, the capacity to reduce interfacial tension at the oil–water interface strongly correlates with the emulsion’s stabilizing properties [68].

### 3.7. Incorporation of WMR, WMRP, and WMRH in Bread Preparation

#### 3.7.1. Chemical and Alveograph Characterization of Wheat Flours

Table 4 illustrates the chemical compositions of the wheat flour of high bread-making quality (FHBM) utilized as a positive control and the wheat flour of low bread-making quality (FLBM). The results indicate significant differences in the chemical compositions of the two flours, FHBM and FLBM. The FHBM demonstrated the highest moisture content (13.19 ± 0.03%). Furthermore, its protein (9.00 ± 0.01%) and fiber (0.50 ± 0.08%) contents were notably higher (*p* < 0.05) compared to those of the FLBM, which measured 8.40 ± 0.08% and 0.10 ± 0.04%, respectively. The FLBM exhibited the highest contents of starch (77.00 ± 0.29%) and minerals (0.36 ± 0.01%), while the lowest levels (73.47 ± 0.10% and 0.33 ± 0.01%, respectively) were detected in the FHBM. However, no significant differences (*p* > 0.05) were noted in the fat contents between the FHBM (0.68 ± 0.10%) and FLBM (0.59 ± 0.04%). The two most prominent components of relevance in wheat flour are starch and storage proteins [69]. When wheat flour starch granules are hydrated, they absorb water and swell, retaining up to half of their dry weight in water. This hydration process causes the starch granules to extend somewhat, but reversibly. Additionally, when combined with gluten in a mixture, a solid network is formed. This network traps the air and gases produced during fermentation in the dough structure during baking and cooling. This trapped gas helps to leaven the bread, giving it a light and airy texture, while also preventing it from collapsing. Therefore, the communication between starch and gluten in the dough plays a crucial role in creating the structure and texture of bread [70].

Table 4 displays the alveograph characteristics of wheat flour doughs assessed using the Chopin alveograph. The FHBM, serving as the positive control, exhibited high deformation work (220 ± 2.87 × 10^−4^ J) and a high P/L ratio (elastic resistance and extensibility balance of flour dough) of 0.85 ± 0.02. Conversely, the FLBM wheat flour dough displayed lower values for deformation work (156 ± 7.49 × 10^−4^ J) and P/L (0.70 ± 0.01).

#### 3.7.2. Alveograph Properties of Dough Enriched with WMR, WMRP and WMRH

Table 5 illustrates the impact of adding WMR, WMRP, and WMRH in varying quantities (0.5%, 1%, and 1.5%) on the alveograph parameters of FLBM wheat flour dough. Remarkably, incorporating the WMR, WMRP, and WMRH at concentrations ranging from 0.5% to 1.5% into the FLBM significantly enhanced both the deformation work and the P/L ratio compared to the control (devoid of additives), with values of W = 156 ± 7.49 × 10^−4^ J and P/L = 0.70 ± 0.01. Indeed, the deformation energy increased, reaching a maximum of 182 ± 7.07 × 10^−4^ J, 181 ± 5.51 × 10^−4^ J, and 177 ± 6.45 × 10^−4^ J upon the incorporation of the WMR (1%), WMRP (1%), and WMRH (1.5%), respectively. However, these deformation work values remained lower than that of the FHBM wheat flour dough (220 ± 2.87 × 10^−4^ J). Similarly, the P/L ratio values of the FLBM wheat flour doughs enriched with the WMR, WMRP, and WMRH increased in proportion to the sample’s amount, significantly surpassing both the FLBM wheat flour doughs without additives (0.70 ± 0.01) and the FHBM used as a positive control (0.85 ± 0.02).

These findings demonstrate that watermelon rind powder, soluble polysaccharides, and hemicellulose exert a favorable influence on the alveograph characteristics of wheat flour dough. Notably, the addition of 1% WMR, WMRP, and WMRH to wheat flour with low bread-making qualities enhanced the deformation work by 16%, 15%, and 13%, respectively, and increased the P/L ratio by 42%, 36%, and 38%, respectively. This influence is probably attributable to the interaction between hemicellulose and wheat flour proteins, as detailed in prior studies by Sudha et al. [71]. Additionally, the synergy between the soluble and insoluble fractions interacts with other compounds within the watermelon rind powder, such as proteins, lipids, and polyphenols. Therefore, the incorporation of dietary fibers in bakery items can modify both the alveograph characteristics of dough and the textural properties of the baked products [21]. Additionally, the formation of dough stands as a crucial phase in the processing of flour-based products. A well-established continuous network of wheat gluten imparts the dough with both viscosity and elasticity. Studies indicate that the quality of dough is directly influenced by the structure of this gluten network. Indeed, researchers have explored the impact of dietary fibers on dough properties and noted that the addition of dietary fibers could both enhance and impair the gluten network structure [72,73]. This behavior might be attributable to variations in the type, structure, size, and quantity of the added dietary fibers [74].

### 3.8. Bread Characterization

**Figure 6:**
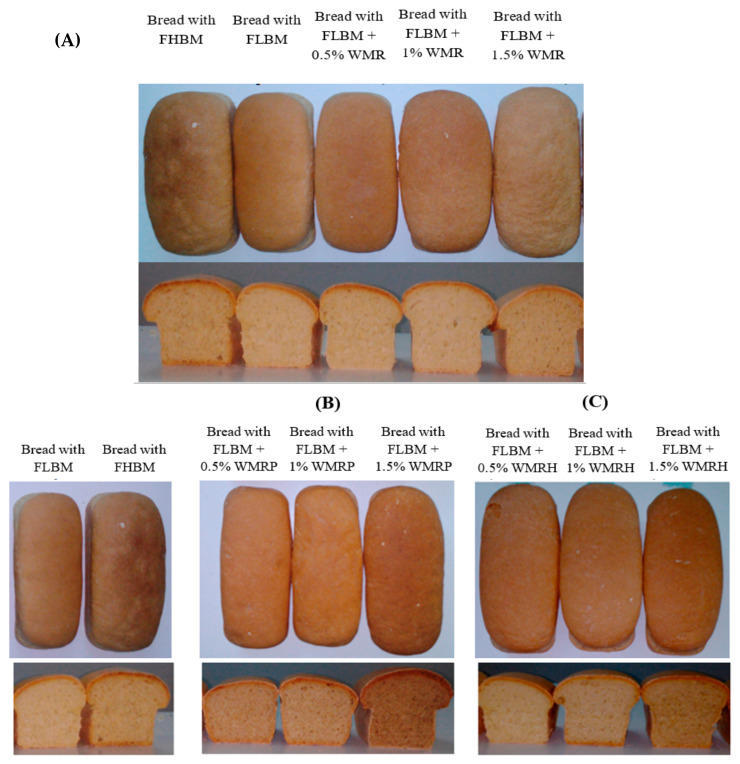
Bread photos (front view and cross-section) prepared with FLBM, FHBM, FLBM supplemented with 0.5%, 1%, and 1.5 of WMR (**A**); FLBM supplemented with 0.5%, 1%, and 1.5 of WMRP (**B**); and FLBM supplemented with 0.5%, 1%, and 1.5 of WMRH (**C**).

Doughs enriched with varying amounts (0.5%, 1%, and 1.5%) of WMR, WMRP, and WMRH were baked, and their properties were compared to those made solely with FLBM and FHBM. Images of the bread are displayed in Figure 6, demonstrating that the visual appearance aligns with the alveograph characteristics of the dough. Certainly, the appearance of the bread enriched with WMR at concentrations of 0.5%, 1%, and 1.5% displayed a visually appealing aspect compared to that made solely with FLBM, showcasing a similar appearance to that of FHBM. Additionally, the bread prepared with FLBM enriched with WMRP (1.5%) or WMRH (1% and 1.5%) exhibited greater visual appeal and higher volume compared to the reference bread made with FLBM. Bread quality assessment encompassed specific volume, texture, and color measurements. Differences in water-holding capacity (WHC) among the bread formulations can significantly impact the appearance and texture of the bread. Higher WHC generally results in a moister, softer bread crumb, giving the bread a less dry appearance and a more desirable texture. On the other hand, lower WHC may lead to drier bread, resulting in a firmer or even crumbly texture. This variation in WHC can affect the bread’s hardness and visual appeal, with formulations retaining more moisture typically producing softer and visually smoother bread. This result was attributed to the higher water-binding capacity of WMR (12 ± 0.3 g/g), which prevents water loss during storage. Moreover, the hydrogen bonds between WMR and starch would also contribute to the delay of the starch retrogradation.

#### 3.8.1. Analysis of Bread Volume

Loaf volume stands as a crucial bread characteristic as it quantitatively assesses baking performance [20,75]. In most bakery products, there exists a correlation between the dough weight and the resulting loaf volume, which contributes to the achievement of the most desirable texture [35,76]. Table 6 presents the specific volume of bread prepared using FLBM, FHBM, and FLBM enriched with 0.5%, 1%, and 1.5% WMR, WMRP, and WMRH. The incorporation of the WMR, WMRP, and WMRH powders into the wheat flour affected the bread’s length, width, and height. Indeed, the length of the bread containing watermelon rind powder and soluble polysaccharides at concentrations of 0.5% and 1% exhibited a notably higher value (*p* < 0.05) compared to the control bread without any additives. However, the length of the bread with 0.5% and 1% hemicellulose was similar (21.50 ± 0.28 cm and 21.50 ± 0.07 cm) and closely resembled that of the control bread (21.50 ± 0.42 cm). Furthermore, the width and height of the bread incorporating the WMRs and their derivatives were notably greater compared to the bread without any additives. However, the bread fortified with 0.5% WMRH maintained similar width and height (11.50 ± 0.25 cm and 7.50 ± 0.45, respectively) to the control bread (11.70 ± 0.21 cm and 7.60 ± 0.28, respectively). Furthermore, the specific volume of the bread expanded with the addition of additives. Specifically, the bread volume increased from 3.5 ± 0.01 cm^3^ in the bread without additives to 3.75 ± 0.04 cm^3^/g and to 3.75 ± 0.05 cm^3^/g with 1% WMR and 1.5% WMRH, respectively. However, the bread fortified with varying levels of WMRP (0.5%, 1%, and 1.5%) displayed the highest specific volumes, measuring 3.75 ± 0.05 cm^3^/g, 3.80 ± 0.02 cm^3^/g, and 3.80 ± 0.03 cm^3^/g, respectively. These specific volumes are notably (*p* < 0.05) greater than those of unenriched bread (3.50 ± 0.01 cm^3^/g) but comparable to the specific volume of bread made with the reference flour (3.70 ± 0.02 cm^3^/g). Similar findings were reported by Blibech et al. [20], indicating that the incorporation of galactomannans in bread formulation enhanced its specific volume.

The positive impact of emulsifiers on bread volume is primarily due to their ability to enhance dough strength by forming liquid films with a lamellar structure at the interface between gluten strands and starch granules [77]. This structural enhancement can be attributed to the unique properties of emulsifiers, particularly their amphiphilic nature. Emulsifiers, which possess both hydrophilic and hydrophobic characteristics, interact with the gluten network in a way that strengthens it. Specifically, anionic emulsifiers interact with the hydrophobic regions of gluten proteins, while also forming hydrogen bonds with the amino groups of glutamines [77]. This interaction significantly enhances the strength of the dough, which is crucial for better retention of carbon dioxide during the oven spring. The stronger dough structure allows improved gas retention, leading to a higher loaf volume in the finished bread [78]. However, it is important to note that not all emulsifiers have the same effect. For instance, the addition of lecithin, another type of emulsifier, has been found to decrease the stability of wheat flour dough, potentially leading to less desirable baking outcomes [77].

Depending on the fiber source and degree of supplementation, adding fiber to bread can either increase or decrease the loaf volume. In fact, bread enhanced with wheat bran [79] or β-glucans [80] has a lower loaf volume and a firmer crumb. Nonetheless, bread enhanced with gums, like almond gum, which has 72.7 ± 1.5% fibers, displayed a higher loaf volume. The bread volume dropped at concentrations of more than 2% almond gum [81].

#### 3.8.2. Textural Properties of Bread

Table 7 presents the texture profile analysis (TPA) values of the bread made from FLBM, FHBM, and FLBM enriched with WMR, WMRP, and WMRH. The findings demonstrate significant enhancement in the textural properties of the breads supplemented with WMR, WMRP, and WMRH compared to those made with FLBM. The hardness of the bread supplemented with 1% WMR notably decreased by 18% (*p* < 0.05) compared to the control bread made with FLBM. Moreover, the hardness of the breads supplemented with the two saccharide fractions, WMRP and WMRH, also significantly decreased (*p* < 0.05) compared to the control bread made with FLBM. Bouaziz et al. [19] highlighted that incorporating almond gum in the bread formulation notably decreased the hardness compared to control bread without almond gum. Similarly, earlier findings by Della Valle [82] showed that high concentrations of fiber adversely impacted the textural quality of the product. Additionally, it has been established that bread hardness results from interactions between gluten and fibrous materials [83]. Moreover, the cohesiveness, springiness, and adhesiveness parameters of the bread enriched with WMR and WMRP displayed notable increases (*p* < 0.05), correlating with the sample concentrations. The values were 0.2 ± 0.01 for cohesiveness, 16 ± 4.7 for springiness, and 0.7 ± 0.32 for adhesiveness in the bread fortified with 1% WMR. Similarly, the bread fortified with 1% WMRP demonstrated values of 0.2 ± 0.06 for cohesiveness, 26 ± 1.58 mm for springiness, and 2 ± 0.41 for adhesiveness.

#### 3.8.3. Analysis of Bread Color

The bread color results, as presented in Table 8, indicate non-significant differences (*p* > 0.05) in crust lightness between the breads enriched with 0.5% (L* = 75.00 ± 4.29) and 1% (L* = 72.50 ± 1.63) WMR and the control bread made with FLBM (L* = 74.00 ± 1.50). With the increased level of watermelon rind powder (1.5%), the L* value of the crust decreased notably (from L* = 74.00 ± 1.50 in the control to 65.50 ± 0.43 in the bread prepared with 1.5% WMR). This alteration might stem from heightened non-enzymatic browning when wheat flour is substituted by fibers with distinct sugar compositions [45]. However, the lightness of both the crust and crumb significantly decreased (*p* < 0.05) subsequent to the addition of WMRP and WMRH in the bread formulation in comparison to the control bread (L* of crust = 74.00 ± 1.50 and L* of crumb = 81.50 ± 1.54) and the reference bread (L* of crust = 65.00 ± 2.61 and L* of crumb = 78.00 ± 1.91). Certainly, the lightness (L*) values stood at 63.00 ± 2.70, 61.00 ± 1.79, and 60.00 ± 1.87 for the crust, and 78.00 ± 0.48, 77.00 ± 1.64, and 72.50 ± 1.32 for the crumb of the bread made with FLBM supplemented with 0.5%, 1%, and 1.5% WMRP, respectively. Similarly, as the amount of the WMRH increased, the L* values of the crust decreased (from 74.00 ± 1.50 in the control to 68.00 ± 1.31, 61.00 ± 0.14, and 58.00 ± 0.72 in the bread prepared with 0.5%, 1%, and 1.5% WMRH, respectively) and the L* values of the crumb also decreased (from 81.5 ± 1.54 in the control to 76.00 ± 1.14, 75.00 ± 0.49 and 65.0 ± 0.18 in the bread enriched with 0.5%, 1%, and 1.5% WMRH, respectively). The incorporation of WMR (1% and 1.5%), WMRP (0.5% to 1.5%), and WMRH (1% and 1.5%) led to a notable increase in the redness (a*) values of both the crust and crumb, resulting in darker shades compared to the control bread (*p* < 0.05).

Additionally, the significant increase in the crumb darkness of the bread fortified with WMRP compared to that of the bread prepared with hemicellulose (WMRH) is directly related to the (a*) values of these fractions. In fact, the redness of the WMRP (a* = 4 ± 0.09) was significantly (*p* < 0.05) more intense than that of the WMRH (a* = 3 ± 0.05) (Table 8). Concerning the yellowness (b*), the crumb of the bread prepared with flour substituted with WMR, WMRP, and WMRP powders showed higher b* values than those of the control. This parameter was not significantly changed in the crust of the bread fortified with WMRP at the different doses compared to the control bread. Conversely, as the amount of the WMRH increased, the b* values of the crust decreased (from b* = 20 ± 0.59 in the control bread to 20.5 ± 0.77, 21 ± 0.17 and to 21 ± 0.30 in the bread prepared with 0.5%, 1%, and 1.5% WMRH, respectively).

## 4. Conclusions

Watermelon rinds (WMRs) were chemically characterized, revealing elevated levels of fiber and protein. These raw materials and their derivatives, particularly the water-soluble polysaccharides (WMRPs) and hemicellulose (WMRH), exhibited an interesting water-holding capacity, fat-binding ability, and potent emulsifying and foaming capabilities. Incorporating WMR, WMRP, and WMRH at concentrations ranging from 0.5 to 1.5% into wheat flour enhances the rheological characteristics of the dough by augmenting the deformation energy and the configuration curve ratio. Enriching wheat flour with WMR notably amplified the bread’s volume and texture in comparison to its derivatives, WMRP and WMRH. Indeed, bread enriched with WMR at a 1% concentration received notably higher appreciation (*p* < 0.05) from the panelists compared to the other bread variations. Based on these findings, it appears plausible to consider that WMRs, followed by WMRP and WMRH, possess favorable qualities for application as a baking enhancer.

## Figures and Tables

**Table 1:** Chemical composition of watermelon rind powder.

Component (g/100 g)	Average Content
Moisture	13.00 ± 0.74
Proteins	17.00 ± 0.13
Fat	2.00 ± 0.14
Ash	2.00 ± 0.56
Total carbohydrate	66.00 ± 2.2
Total fiber	55.00 ± 1.8
Soluble fiber	9.00 ± 0.72
Insoluble fiber	46.00 ± 2.6
Cellulose	21.00 ± 0.30
Hemicellulose	25.00 ± 2.3
**Minerals (mg/100 g)**	
K	601 ± 17
Ca	346 ± 12
Na	287 ± 10
Mg	124 ± 6
Zn	3 ± 0.03
Mn	0.7 ± 0.01
Fe	1.5 ± 0.02
Cu	<0.001

**Table 2:** Water activity and color characteristics of WMR, WMRP, and WMPH and wheat flour (Ff). The data are the mean standard deviation values of five replicates. Different lowercase letters indicate significant differences (*p* < 0.05) between the different powders.

Samples	Color			aw
	L*	a*	b*	
WMR	80.00 ± 0.13 ^a^	0.80 ± 0.11 ^a^	21.00 ± 0.22 ^a^	0.30 ± 0.02 ^a^
WMRP	45.00 ± 0.22 ^b^	7.50 ± 0.12 ^b^	15.50 ± 0.55 ^b^	0.40 ± 0.02 ^b^
WMRH	40.00 ± 0.75 ^b^	4.00 ± 0.20 ^c^	7.00 ± 0.06 ^c^	0.30 ± 0.06 ^a^
Wheat Flour (Ff)	98.00 ± 0.29 ^c^	−1.50 ± 0.01 ^d^	12.00 ± 0.71 ^d^	0.50 ± 0.08 ^c^

**Table 3:** Water-holding and fat-binding capacities of WMR, WMRP, and WMRH. Values are given as mean ± SD from triplicate determinations. Different superscripts in the same line mean the significant differences (*p* < 0.05).

	WMR	WMRP [37]	WMRH
Water-holding capacity (g H_2_O/g sample)	12.00 ± 0.3 ^a^	2.00 ± 0.2 ^b^	2.00 ± 0.2 ^b^
Fat-binding capacity g oil/g sample)	2.00 ± 0.1 ^a^	4.00 ± 0.1 ^b^	4.00 ± 0.1 ^b^

**Table 4:** Chemical characterization of wheat flours and alveograph parameters of dough of FHBM and FLBM. The data are the average ± standard deviation of three replicates. The values not sharing the same letter (a–b) within a column are significantly different (*p* < 0.05).

Chemical Characteristics of Wheat Flours								
	Moisture (%)		Protein (%)		Fiber (%)	Fat (%)	Starch (%)	Ash (%)
**FHBM**	13.00 ± 0.03 ^b^		9.00 ± 0.01 ^b^		0.50 ± 0.08 ^b^	0.70 ± 0.10 ^a^	74.00 ± 0.10 ^b^	0.40 ± 0.01 ^b^
**FLBM**	11.00 ± 0.06 ^a^		8.50 ± 0.08 ^a^		0.10 ± 0.04 ^a^	0.60 ± 0.04 ^a^	77.00 ± 0.29 ^a^	0.40 ± 0.01 ^a^
**Alveograph parameters of dough**								
		W (10–4 J)		P (mm H_2_O)		L (mm)		P/L
**Dough of FHBM**		220 ± 2.87 ^a^		75.00 ± 2.83 ^a^		87.00 ± 2.12 ^a^		0.85 ± 0.02 ^a^
**Dough of FLBM**		156 ± 7.49 ^b^		55.00 ± 3.53 ^b^		80.00 ± 4.10 ^b^		0.70 ± 0.01 ^b^

**Table 5:** The alveograph characteristics of FLBM wheat flour dough enriched with different amounts of WMR, WMRP, and WMRH. The data are the average ± standard deviation of three replicates. The values not sharing the same letter (a–f) within a column are significantly different (*p* < 0.05).

Dough	W (10^−4^ J)	P (mm H_2_O)	L (mm)	P/L
**FHBM**	220 ± 2.87 ^e^	74.00 ± 2.83 ^d^	86.50 ± 2.12 ^e^	0.85 ± 0.02 ^e^
**FLBM**	156 ± 7.49 ^ad^	55.00 ± 3.53 ^a^	80.00 ± 4.10 ^a^	0.70 ± 0.01 ^a^
**FLBM + WMR**				
**0.5%**	171 ± 11.31 ^b^	69.00 ± 1.30 ^b^	68.00 ± 1.83 ^b^	1.00 ± 0.03 ^b^
**1%**	182 ± 7.07 ^c^	69.50 ± 1.70 ^b^	71.72 ± 2.12 ^c^	0.90 ± 0.16 ^b^
**1.5%**	162 ± 3.14 ^a^	75.00 ± 1.23 ^d^	55.25 ± 1.76 ^d^	1.50 ± 0.01 ^c^
**FLBM + WMRP**				
**0.5%**	178 ± 4.52 ^c^	58.00 ± 2.25 ^b^	69.50 ± 2.54 ^b^	0.80 ± 0.04 ^bc^
**1%**	181 ± 5.51 ^c^	63.50 ± 1.94 ^c^	68.00 ± 2.12 ^b^	0.90 ± 0.05 ^de^
**1.5%**	182 ± 4.67 ^c^	76.50 ± 2.13 ^d^	66.50 ± 3.71 ^b^	1.20 ± 0.09 ^f^
**FLBM + WMRH**				
**0.5%**	156 ± 5.31 ^a^	53.00 ± 1.26 ^a^	69.50 ± 2.80 ^b^	0.80 ± 0.02 ^ab^
**1%**	166 ± 4.24 ^b^	58.00 ± 2.19 ^b^	69.50 ± 2.12 ^b^	0.80 ± 0.04 ^bc^
**1.5%**	177 ± 6.45 ^c^	62.50 ± 1.40 ^c^	66.50 ± 2.82 ^b^	0.95 ± 0.05 ^e^

**Table 6:** Specific volumes of bread prepared with FLBM, FHBM, and FLBM supplemented with 0.5%, 1%, and 1.5 of WMR, WMRP, and WMRH. The data are the average ± standard deviation of three replicates. The values not sharing the same letter (a–c) within a column are significantly different (*p* < 0.05).

Bread	Length (cm)	Width (cm)	Height (cm)	Specific Volume (cm^3^/g)
**Bread with FHBM**	21.90 ± 0.14 ^b^	12.35 ± 0.07 ^b^	8.45 ± 0.07 ^b^	3.7 ± 0.02 ^b^
**Bread with FLBM**	21.50 ± 0.42 ^a^	11.65 ± 0.21 ^a^	7.58 ± 0.28 ^a^	3.5 ± 0.01 ^a^
**Bread with FLBM + WMR**				
**0.5%**	21.90 ± 0.14 ^b^	12.20 ± 0.48 ^b^	8.50 ± 0.42 ^b^	3.50 ± 0.01 ^a^
**1%**	22.00 ± 0.42 ^b^	12.20 ± 0.28 ^b^	8.40 ± 0.56 ^b^	3.75 ± 0.04 ^c^
**1.5%**	21.85 ± 0.07 ^b^	12.15 ± 0.07 ^b^	8.15 ± 0.21 ^b^	3.70 ± 0.04 ^b^
**Bread with FLBM + WMRP**				
**0.5%**	22.15 ± 0.21 ^b^	12.50 ± 0.14 ^b^	8.20 ± 0.14 ^abc^	3.75 ± 0.05 ^b^
**1%**	22.00 ± 0.31 ^ab^	12.00 ± 0.14 ^c^	8.35 ± 0.77 ^bc^	3.80 ± 0.02 ^b^
**1.5%**	21.95 ± 0.42 ^ab^	12.30 ± 0.26 ^bc^	8.65 ± 0.21 ^c^	3.80 ± 0.03 ^b^
**Bread with FLBM + WMRH**				
**0.5%**	21.50 ± 0.28 ^a^	11.50 ± 0.25 ^a^	7.50 ± 0.45 ^a^	3.50 ± 0.06 ^a^
**1%**	21.55 ± 0.07 ^a^	12.10 ± 0.14 ^c^	7.65 ± 0.35 ^ab^	3.50 ± 0.04 ^a^
**1.5%**	22.00 ± 0.45 ^ab^	12.50 ± 0.21 ^b^	8.55 ± 0.49 ^c^	3.75 ± 0.05 ^b^

**Table 7:** The effect of WMR, WMRP, and WMRH addition on the textural properties (hardness, cohesiveness, adhesiveness, and springiness) of bread. The data are the average ± standard deviation of three replicates. The values not sharing the same letter (a–f) within a column are significantly different (*p* < 0.05).

Bread	Hardness (N)	Cohesiveness	Springiness (mm)	Adhesiveness (N)
**Bread with FHBM**	4.00 ± 0.38 ^e^	0.09 ± 0.01 ^a^	9.00 ± 0.74 ^a^	0.50 ± 0.15 ^b^
**Bread with FLBM**	5.00 ± 0.07 ^a^	0.09 ± 0.01 ^a^	9.00 ± 0.71 ^a^	1.00 ± 0.04 ^a^
**Bread with FLBM + WMR**				
**0.5%**	5.00 ± 0.10 ^ad^	0.10 ± 0.01 ^a^	9.00 ± 1.44 ^a^	0.50 ± 0.15 ^b^
**1%**	4.00 ± 0.05 ^be^	0.20 ± 0.01 ^b^	16.00 ± 4.7 ^b^	0.70 ± 0.32 ^b^
**1.5%**	4.50 ± 0.42 ^bd^	0.20 ± 0.05 ^b^	19.00 ± 1.95 ^b^	0.70 ± 0.27 ^b^
**Bread with FLBM + WMRP**				
**0.5%**	4.00 ± 0.54 ^b^	0.10 ± 0.03 ^ac^	9.00 ± 0.40 ^a^	0.40 ± 0.04 ^bf^
**1%**	4.00 ± 0.39 ^bc^	0.2 ± 0.06 ^b^	26.00 ± 1.58 ^b^	2.00 ± 0.41 ^c^
**1.5%**	3.50 ± 0.51 ^b^	0.06 ± 0.01 ^a^	5.00 ± 0.29 ^c^	0.20 ± 0.02 ^b^
**Bread with FLBM + WMRH**				
**0.5%**	4.50 ± 0.12 ^c^	0.20 ± 0.09 ^b^	19.00 ± 0.79 ^d^	1.50 ± 0.09 ^d^
**1%**	4.50 ± 0.65 ^c^	0.10 ± 0.01 ^ac^	11.00 ± 0.42 ^e^	0.50 ± 0.09 ^bf^
**1.5%**	4.50 ± 0.53 ^c^	0.15 ± 0.04 ^bc^	15.00 ± 0.97 ^f^	0.80 ± 0.05 ^e^

**Table 8:** The effect of WMR, WMRP, and WMRH addition on the color characteristics of bread. The data are the average ± standard deviation of three replicates. The values not sharing the same letter (a–g) within a column are significantly different (*p* < 0.05).

	Bread	Lightness L*	Redness a*	Yellowness b*
**Crust**	**Bread with FHBM**	65.00 ± 2.61 ^b^	10.00 ± 0.93 ^d^	32.00 ± 0.51 ^d^
	**Bread with FLBM**	74.00 ± 1.50 ^a^	7.00 ± 0.05 ^a^	32.00 ± 0.96 ^ad^
	**Bread with FLBM + WMR**			
	**0.5%**	75.00 ± 4.29 ^a^	6.00 ± 0.82 ^b^	32.00 ± 0.12 ^ad^
	**1%**	72.50 ± 1.63 ^a^	9.00 ± 0.29 ^c^	34.50 ± 0.90 ^b^
	**1.5%**	65.50 ± 0.43 ^b^	9.00 ± 0.06 ^cd^	31.50 ± 0.01 ^ac^
	**Bread with FLBM + WMRP**			
	**0.5%**	63.00 ± 2.70 ^bf^	10.00 ± 0.69 ^b^	32.00 ± 0.83 ^a^
	**1%**	61.00 ± 1.79 ^bc^	10.50 ± 0.55 ^b^	31.50 ± 0.43 ^a^
	**1.5%**	60.00 ± 1.87 ^ce^	10.50 ± 0.27 ^b^	32.00 ± 0.29 ^a^
	**Bread with FLBM + WMRH**			
	**0.5%**	68.00 ± 1.31 ^d^	8.00 ± 0.98 ^a^	30.50 ± 0.87 ^b^
	**1%**	61.00 ± 0.14 ^bc^	9.00 ± 0.01 ^c^	28.00 ± 0.01 ^c^
	**1.5%**	58.00 ± 0.72 ^e^	10.00 ± 0.15 ^b^	29.00 ± 0.65 ^c^
**Crumb**	Bread with FHBM	78.00 ± 1.91 ^bc^	−0.60 ± 0.12 ^b^	22.00 ± 0.41 ^cd^
	**Bread with** **FLBM**	81.50 ± 1.54 ^a^	−1.00 ± 0.24 ^a^	20.00 ± 0.59 ^a^
	**Bread with FLBM + WMR**			
	**0.5%**	79.00 ± 0.32 ^b^	−0.40 ± 0.51 ^b^	22.00 ± 0.83 ^bd^
	**1%**	77.50 ± 0.96 ^c^	−0.70 ± 0.13 ^b^	21.00 ± 0.63 ^b^
	**1.5%**	77.00 ± 1.02 ^c^	−0.70 ± 0.01 ^b^	21.00 ± 0.28 ^b^
	**Bread with FLBM + WMRP**			
	**0.5%**	78.00 ± 0.48 ^bc^	1.50 ± 0.06 ^b^	23.50 ± 0.01 ^b^
	**1%**	77.00 ± 1.64 ^bc^	3.00 ± 0.04 ^c^	23.50 ± 0.26 ^b^
	**1.5%**	72.50 ± 1.32 ^e^	4.00 ± 0.09 ^d^	25.00 ± 0.20 ^c^
	**Bread with FLBM + WMRH**			
	**0.5%**	76.00 ± 1.14 ^cd^	1.00 ± 0.20 ^e^	20.50 ± 0.77 ^ad^
	**1%**	75.00 ± 0.49 ^de^	2.50 ± 0.06 ^f^	21.00 ± 0.17 ^de^
	**1.5%**	65.00 ± 0.18 ^f^	3.00 ± 0.05 ^g^	21.00 ± 0.30 ^e^

## Data Availability

The original contributions presented in the study are included in the article, further inquiries can be directed to the corresponding author.
